# The Neural Cascade of Attentional Control

**DOI:** 10.1371/journal.pbio.0050027

**Published:** 2007-01-02

**Authors:** Janelle Weaver

In our complex and multifaceted world, our senses are continuously bombarded with much more information than our brains can fully process. Attention is a critical cognitive function that allows us to select for detailed analysis only the most relevant information at any given moment. The crucial nature of attention is clearly evident in the game “Where's Waldo.” Picking out the odd, bespectacled man wearing the familiar red-and-white striped shirt and hat amidst an array of distracting objects that contain similar colors and patterns is astonishingly difficult. Only by directing our attention or our eyes sequentially to many different spatial locations can we eventually find Waldo. However, the basic neural mechanisms that allow us to focus our attention on specific locations in our visual field, even in uncluttered scenes, are not well understood.

Previous functional neuroimaging studies have revealed a network of frontal and parietal brain regions that, along with the visual cortex, show enhanced activity during visual-spatial attention. It has been hypothesized that this frontal-parietal network controls attention by sending “top-down” signals that “bias” activity in specific regions of visual cortex. This biasing activity is, in turn, thought to facilitate the accurate, rapid processing of information in attended regions of space. However, these neuroimaging studies were unable to elucidate the temporal cascade of neural events associated with the control of attention—critical information for understanding how different brain regions coordinate their actions as succeeding stages of attentional control unfold.

In this study, Tineke Grent-‘t-Jong and Marty Woldorff set out to discover both the timing and location of brain activations in the attentional control network that underlie our ability to direct our visual spatial attention. To do this, the authors combined two complementary methods: event-related potentials (ERPs), which measure electrical changes in the brain with scalp electrodes and thus provide very precise timing information on brain activity triggered by sensory or cognitive events, and functional magnetic resonance imaging (fMRI), which localizes event-triggered brain activations by measuring associated changes in blood flow. This innovative study surmounted previous obstacles to the study of attentional control by including important control conditions and by using the exact same experimental paradigm and task contrasts (comparisons of patterns of activation elicited by different tasks) for both methodologies. This multimethodological approach enabled the authors to obtain measures of cognitive brain activity with good temporal and spatial resolution and thereby gain a more complete understanding of the neural mechanisms underlying top-down attentional control.

The authors used a spatial cueing paradigm in which participants kept their visual gaze directed toward the center of a computer screen. A series of 4-second trials were presented, with an instructional letter cue displayed at the beginning of each trial. On most trials, the letter cue (“L” or “R”) instructed them to covertly attend (that is, without moving their eyes) to either the lower left or the lower right portion of the screen to try to detect the possible brief appearance of a faint dot at the cued location. However, the authors included an important control condition: on some trials, the cue (“P”) instructed the subject to not orient their attention to a particular location, as no faint dot would appear during that trial. By incorporating both of these interpret-cue trials and attention-orienting trials, Grent-‘t-Jong and Woldorff were able to separate the activations related to spatial orienting from those associated with general cue processing that were not related to orienting.

Both the ERP and fMRI results revealed that some activations were elicited by both the interpret cues and the attentional-orienting cues, and some were associated only with attentional orienting. More specifically, the ERPs showed that during the first 400 ms, the attend-cue and interpret-cue trials produced similar patterns of activity, which the fMRI indicated was associated with the more lateral regions of frontal and parietal cortex. After 400 ms, the response to the interpret cues quickly returned to baseline, whereas the attend cues elicited additional, widespread waves of orienting-specific activity that continued for hundreds of milliseconds. Moreover, the fMRI confirmed that this orienting-related activity was associated with greater activation specifically in the more medial portions of the frontal and parietal cortex. These results indicate that lateral parts of the frontal-parietal network play a role in the initial general cue processing and interpretation, whereas the more medial frontal-parietal regions function more specifically in the subsequent orienting of spatial attention.

The authors then set out to determine the relative timing of the medial frontal and parietal activations underlying this attentional-orienting process. To do this, the authors used modeling procedures to relate these orienting-specific regions delineated by the fMRI to the corresponding orienting-specific ERP activity. This analysis indicated that the initial part of this ERP orienting activity (from 400–700 ms) derived mainly from the medial frontal regions. Starting at around 700 ms, however, the analyses indicated that both the frontal and parietal medial regions contributed to the ERP activity patterns, indicating that the orienting-related frontal activation preceded the parietal activation by several hundred milliseconds. These frontal and parietal activations were then followed shortly after by enhanced, biasing-related activity in specific regions of the visual cortex, before the arrival of any target stimuli in the attended region of space.

Altogether, these results reveal the systems-level timing and sequence of neural events underlying the voluntary orienting of spatial attention. More specifically, these results indicate that voluntary attentional orienting is initiated by the more medial portions of the frontal cortex, which then recruit medial parietal areas a few hundred milliseconds later. These regions then act in concert to sustain attention and provide a biasing signal to the specific regions of the visual cortex that process stimuli in the attended region of space. This biasing-related activity is in turn thought to reflect a neurophysiological process that facilitates the processing in the visual cortex of visual stimuli in the attended location.

**Figure:**
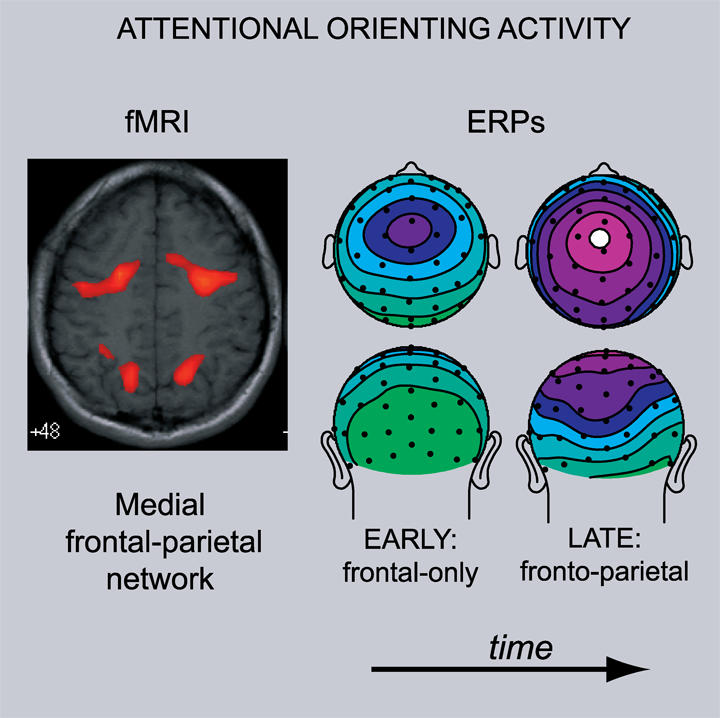
Within the medial frontal-parietal attentional control network (fMRI activations, left column), the frontal areas (ERPs, middle column) activate earlier than the parietal areas (ERPs, right column).

